# Foot-and-mouth disease-associated myocarditis is age dependent in suckling calves

**DOI:** 10.1038/s41598-024-59324-9

**Published:** 2024-05-04

**Authors:** Pankaj Deka, Sangeeta Das, Ritam Hazarika, Ray Kayaga, Biswajit Dutta, Abhijit Deka, Utpal Barman, Rofique Ahmed, Nazrul Islam, Mihir Sarma, Ilakshy Deka, Manoranjan Rout, Krishna Sharma, Rajeev K. Sharma

**Affiliations:** 1 College of Veterinary Science, Assam Agricultural University, Khanapara, Guwahati, Assam 781022 India; 2 Tanzania Veterinary Laboratory Agency, 131 Barabara Ya Nelson Mandela, P.O BOX 9254, Temeke, Dar Es Salaam Tanzania; 3 Indian Veterinary Research Institute, Izatnagar, Bareilly, Uttar Pradesh 243122 India; 4 Krishi Vigyan Kendra, Kamrup, Assam Agricultural University, Kahikuchi Campus, Guwahati, 781017 India; 5 ICAR-Directorate of Foot-and-Mouth Disease, International Centre for FMD, Bhubaneswar, Khordha, Odisha 752050 India

**Keywords:** Foot-and-mouth disease, Myocarditis, Suckling calves, Biochemical markers, Tigroid heart, Biochemistry, Microbiology, Biomarkers

## Abstract

Myocarditis is considered a fatal form of foot-and-mouth disease (FMD) in suckling calves. In the present study, a total of 17 calves under 4 months of age and suspected clinically for FMD were examined for clinical lesions, respiratory rate, heart rate, and heart rhythm. Lesion samples, saliva, nasal swabs, and whole blood were collected from suspected calves and subjected to Sandwich ELISA and reverse transcription multiplex polymerase chain reaction (RT-mPCR) for detection and serotyping of FMD virus (FMDV). The samples were found to be positive for FMDV serotype “O”. Myocarditis was suspected in 6 calves based on tachypnoea, tachycardia, and gallop rhythm. Serum aspartate aminotransferase (AST), creatinine kinase myocardial band (CK-MB) and lactate dehydrogenase (LDH), and cardiac troponins (cTnI) were measured. Mean serum AST, cTn-I and LDH were significantly higher (P < 0.001) in < 2 months old FMD-infected calves showing clinical signs suggestive of myocarditis (264.833 ± 4.16; 11.650 ± 0.34 and 1213.33 ± 29.06) than those without myocarditis (< 2 months old: 110.00 ± 0.00, 0.06 ± 0.00, 1050.00 ± 0.00; > 2 months < 4 months: 83.00 ± 3.00, 0.05 ± 0.02, 1159.00 ± 27.63) and healthy control groups (< 2 months old: 67.50 ± 3.10, 0.047 ± 0.01, 1120.00 ± 31.62; > 2 months < 4 months: 72.83 ± 2.09, 0.47 ± 0.00, 1160.00 ± 18.44). However, mean serum CK-MB did not differ significantly amongst the groups. Four calves under 2 months old died and a necropsy revealed the presence of a pathognomic gross lesion of the myocardial form of FMD known as “tigroid heart”. Histopathology confirmed myocarditis. This study also reports the relevance of clinical and histopathological findings and biochemical markers in diagnosing FMD-related myocarditis in suckling calves.

## Introduction

Foot-and-mouth disease (FMD) is a highly contagious vesicular disease of cloven-hoofed animals. The disease is caused by the FMD virus (FMDV) within the genus *Aphthovirus* of the *Picornaviridae* family. The causative agent is a non-enveloped virus with an icosahedral capsid composed of 60 copies each of four structural proteins (VP1, VP2, VP3, and VP4). The genome is a single-stranded positive-sense RNA of approximately 8500 nucleotides. Seven antigenically distinct serotypes (O, A, C, Asia 1, South African Territories 1, 2, and 3) of FMDV have been identified serologically^1–3^. It is considered one of the most important livestock diseases worldwide due to its severe socio-economic impacts^4,5^. The disease is characterized by fever, vesicular lesions on the tongue, feet, and teats, and lameness. The severity of clinical signs varies with the strain of the virus, degree of exposure, the host species, and the age and immune status of the animal. However, adult ruminants’ mortality is low but higher in young animals due to acute myocarditis^5–7^. In calves, myocarditis is considered a fatal form of FMD as the affected calves die suddenly without showing any clinical signs^7–9^. In field conditions, diagnosis of FMDV-induced myocarditis is very challenging. It is done based on the history of FMD outbreaks in the region, the incidence of the sudden death of young calves, physical examination, and cardiac auscultation. The estimation of serum cardiac troponin I (cTn I), aspartate aminotransferase (AST), creatinine kinase myocardial band (CK-MB), and lactate dehydrogenase (LDH) have been used as diagnostic biomarkers^9–11^. The release of cTn I and other enzymes into circulation as a response to myocardial injury or damage. Studies report that although the enzymes AST, CK-MB, and LDH are biomarkers for myocardial damage, they lack tissue specificity and sensitivity^12^. On the other hand, cTn I has nearly absolute myocardial tissue specificity and higher sensitivity than those enzymes because cTn I is the only troponin uniquely expressed in the myocardium^11,13,14^. Therefore, cTn I can be considered to be a sensitive marker of myocardial damage as compared to the enzymes.The present study aims to detect and type the FMDV circulating in Assam. This study is also focused on mortality in calves with myocarditis and the relevance of clinical and histopathological findings as well as biochemical markers in FMD diagnosis in suckling calves.

## Materials and methods

### Sampling and study design

Seventeen calves under 4 months of age and suspected clinically of FMD were included in the present study. After a thorough clinical investigation, the calves were divided into two groups based on their age, group A_0_, aging < 2 months (n = 7), and B_0_, aging > 2–< 4 months (n = 10). Alternatively, the calves were divided into three groups, group A_1_ (< 2 months old, n = 6) clinically suggestive of myocarditis, and group A2 (< 2 months old, n = 1) and B_1_ (> 2 months and < 4 months old, n = 10) clinically negative for myocarditis. As control groups, twelve healthy calves were also used, aging < 2 months (n = 6) and > 2–< 4 months (n = 6) and grouped as C and D respectively. Lesion samples (epithelial tissue and vesicular fluid), whole blood, saliva, and nasal swabs were collected from suspected calves for the detection of FMDV. The criteria for the selection of suspected calves depended on characteristic FMD clinical symptoms such as fever, salivation, vesicles in the tongue and mouth, depression, and loss of appetite. Blood samples were collected from both clinically affected and control calves for prognostic study (serum biochemistry analysis and cardiac biomarker study). In addition, four dead calves under 2 months old suspected clinically of myocarditis were included in the study for necropsy and histopathological examination. The sampling period of the study was 2019–2022. This work has been carried out with high biosafety and biosecurity standards in the BSL-3 laboratory, DBT-Advanced Animal Disease Diagnosis and Management Consortium (ADMaC), Guwahati center, Assam Agricultural University, Khanapara—781022, Assam, India. . Four calves under 2 months old suspected clinically of myocarditis died.

### FMDV detection and typing

Sandwich ELISA and reverse transcription multiplex polymerase chain reaction (RT-mPCR) were performed for the detection and serotyping of FMDV in samples collected from FMD suspected calves.

#### Sandwich ELISA

Typing of FMDV in epithelial tissue and vesicular fluid (lesion samples) from suspected infected calves was performed by Sandwich ELISA developed by ICAR-DFMD, Mukteshwar, India. A 10% (W/V) antigen suspension of epithelial tissue was prepared in phosphate-buffered saline (PBS). Briefly, tissue samples were triturated with PBS, and the suspension was mixed with an equal volume of chloroform. After centrifugation at 1000 g for 15 min, the supernatant was collected as antigen and stored at −20 °C for further use. Vesicular fluid was directly used as an antigen for the detection of FMDV.

Type-specific anti-146S FMDV sera raised in rabbits and guinea pigs were used as coating and tracing sera respectively as per the recommendation of ICAR-DFMD, Mukteshwar, India. Rabbit anti-guinea pig IgG-HRP conjugate was used in the assay.

#### Viral RNA extraction and RT-mPCR

Viral RNA was extracted from lesion samples, saliva, nasal swab, and blood collected from FMD suspected calves using a QIAamp Viral RNA kit (Qiagen, Cat No. 52904) according to the manufacturer’s protocol. Extracted RNA was quantified and cDNA synthesis was done immediately using the RevertAid First Strand cDNA Synthesis Kit (Thermo scientific, Cat No. EP0441).

RT-mPCR was carried out according to the procedure described by^15^ and^16^. To identify the virus type, the target partial genes of each prevailing serotype in India (type O, A, and Asia-1) were amplified using the reported primers of^17^. The list of primers used in RT-mPCR is enlisted in Table 1. The PCR products were analyzed on 2% agarose gel electrophoresis using Tris Borate EDTA buffer (1X) buffer stained with ethidium bromide. Using a gel documentation system, a 100-bp DNA ladder was used to identify the amplified product size.

**Table 1:** Primers used for amplification of serotype-specific FMDV by RT-mPCR.

Serotypes of FMDV	Primers	Sequences [5′–3′]
A	DHP15	CAACGGGACGARCAAGTACTC
O	DHP13	GTGACTGAACTGCTTTACCGCAT
Asia-1	DHP9	GACCTGGAGGTYGCGCTTGT
Universal (Reverse sense)	NK61	GACATGTCCTCCTGCATCTG

### Serum biochemistry analysis

The serum AST, CK-MB, and LDH were estimated using a semi-automatic biochemical analyzer (Avantor) with definite respective kits following the manufacturer’s protocol (Benesphera). Cardiac troponins, especially cTn I is the best cardiac biomarker for myocardial damage^11^. The cTn-I values were measured using the specific kit available following the manufacturer’s protocol (Benesphera).

### Necropsy and histopathological examination

All the dead calves were subjected to necropsy. Lesions were observed and heart sample was collected. Further, the heart specimen was fixed in 10% buffered formalin for histopathological examination^18^.

### Statistical analysis

Statistical analysis of the data in the present study was done using GraphPad Prism 9.3.0®. Simple one-way ANOVA followed by Tukey’s post hoc test was performed to determine the association of each serum biochemistry parameter with the clinical signs suggestive of myocarditis.

### Ethical approval

All authors certified that the present work was carried after approval of Institute Animal Ethic Committee (IAEC) and as per the guidelines set by the Committee for the Purpose of Control and Supervision of Experiments on Animals (CPCSEA), Animal Welfare Division, Government of India. The ethical approval for the present study was accorded by the IAEC vide Approval No. 770/GO/Re/S/03/CPCSEA/FVSc/AAU/IAEC/18–19/667 dated 28.12.2018. The study is reported per ARRIVE guidelines.

### Consent to participate

All authors participated voluntarily in the research.

## Results

### Typing of FMDV by Sandwich ELISA

A total of 8 lesion samples (epithelial tissue, n = 7 and vesicular fluid, n = 1) were subjected to Sandwich ELISA for detection of FMDV, of which all samples were found to be positive for serotype “O” (Table 2).

**Table 2:** Typing and detection of FMDV by Sandwich ELISA and RT-mPCR.

Nature of samples	Number of samples tested	Test result	
		Sandwich ELISA [number positive]	RT-mPCR [number positive]
Vesicular fluid	1	FMDV Type O [n = 1]	GD [n = 1]
Epithelial tissue	7	FMDV Type O [n = 7]	GD [n = 7]
Saliva	5	ND	GD [n = 5]
Nasal swab	12	ND	GD [n = 9]
Blood	17	ND	GD [n = 11]

GD: Genome detected, ND: Not done.

### Detection of FMDV by RT-mPCR

**Figure 1:**
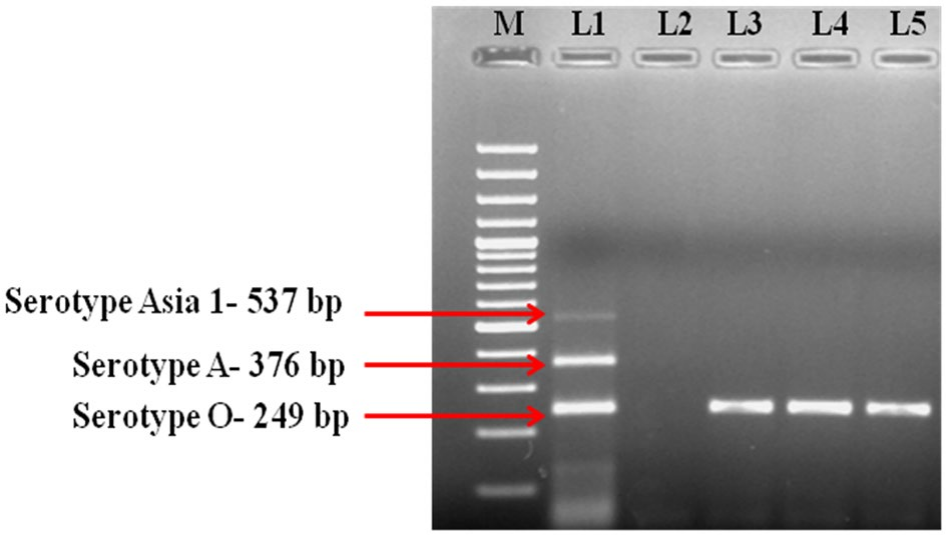
Depicting FMDV serotyping by gel-based RT-mPCR. M: DNA marker (100 bp); Lane 1: Positive control; Lane 2: Negative control; Lane 3–4: Field samples.

RT-PCR was employed on 17 whole blood, 12 nasal swabs, 5 saliva, 7 epithelial tissues, and 1 vesicular fluid sample collected from FMD suspected calves. Of the 42 samples, 33 were found to be positive for FMDV type O (Table 2). Figure 1 indicates the RT-mPCR-based serotype identification where a product size of 249 bp specific for FMDV serotype “O” was amplified in all positive samples.

### Prognostic study

All the samples collected from clinically suspected calves were found to be positive for FMDV type O in Sandwich ELISA or RT-mPCR or both (Fig. 1 and Table 2). The clinical findings recorded in different groups are presented in Table 3. In the anamnesis, clinical findings in (group A_0_: A1 and A2) calves under 2 months old (n = 7) and (group B_0_: B_1_) calves aged > 2– < 4 months (n = 8) included fever (> 40.5 °C), and vesicular lesions on the mouth were noticed in 57.14% and80.00% calves in group A_0_ and B_0_, respectively. All the calves in group B_1_ showed vesicular lesions on the mouth and/or feet. However, none in group B_1_ showed gallop rhythm. Further, in the calves diagnosed as FMD positive with clinical signs suggestive of myocarditis (group A_1_), the clinical symptoms were fever (> 40.5 °C), tachypnea (> 50 breaths per min), tachycardia (> 100 beats per min), and gallop rhythm was noticed in all (100%) and vesicular lesions in 50% calves. In the calves diagnosed as FMD positive with clinical signs negative for myocarditis (group A_2_ and B_1_), fever (> 40.5 °C) was determined in 100% and 80% of calves respectively, and vesicular lesions in all (100%). All calves with clinical signs suggestive of myocarditis were under 2 months old. Four calves under 2 months old suspected clinically of myocarditis died.

**Table 3:** Clinical findings recorded in different groups of calves.

Parameters	FMD positive							Control [n = 12]	
	A_0_ < 2 months age (n = 7)		B_0_ > 2- < 4 months age (n = 10)	A_1_ Suggestive of myocarditis positive (n = 6)	A_2_ Suggestive of myocarditis negative (n = 1)	B_1_ Suggestive of myocarditis negative (n = 10)		C < 2 months age (n = 6)	D > 2- < 4 months age (n = 6)
High temperature [> 40.5 °C ]	+ (n = 7)		+ (n = 8)	+ (n = 6)	+ (n = 1)	+ (n = 8)		–	–
Tachypnea [> 50 breaths per min]	+ (n = 6)		–	+ (n = 6)	–	–		–	–
Tachycardia [> 100 beats per min]	+ (n = 6)		–	+ (n = 6)	–	–		–	–
Gallop rhythm	+ (n = 6)		–	+ (n = 6)	–	–		–	–
Lesions of FMD only on the mouth	+ (n = 4)		+ (n = 8)	+ (n = 3)	+ (n = 1)	+ (n = 8)		–	–
Lesions of FMD only on foot	–		+ (n = 2)	–	–	+ (n = 2)		–	–
Lesions of FMD on both foot and mouth	–		+ (n = 7)	–	–	+ (n = 7)		–	–

### Serum biochemistry analysis

Serum parameters of CK-MB, LDH, AST, and cTn-I were compared amongst myocarditis (group A_1_) and non-myocarditis (group A_2_ and B_1_) with FMD and control groups (Table 4, Supplementary Table 1). Serum biochemical analysis revealed mean serum.

**Table 4:** Serum biochemical findings of calves in different groups.

Parameters	FMD-positive calves			FMD-negative healthy control groups	
	< 2 months old calves (A_0_)		> 2 months < 4 months old calves (B_0_)	< 2 months old calves (C)	> 2 months < 4 months old calves (D)
	Clinical signs suggestive of myocarditis positive (A_1_)	Clinical signs suggestive of myocarditis negative (A_2_)	Clinical signs suggestive of myocarditis negative (B_1_)		
cTn-I [μg/L]	11.65.00^a^ ± 0.34	0.06^**b**^ ± 0.00	0.05^**b**^ ± 0.02	0.047^**b**^ ± 0.01	0.047^**b**^ + 0.00
AST [U/L]	264.83^**a**^ ± 4.16	110.00^**b**^ ± 0.00	83.0 °C ± 3.00	67.50^**d**^ ± 3.10	72.83^**d**^ ± 2.09
CK-MB [U/L]	233.33^**a**^ ± 12.56	210.00^**a**^ ± 0.00	256.00^**a**^ ± 14.47	256.67^**a**^ ± 17.64	240.00^**a**^ ± 14.61
LDH [U/L]	1213.33^**a**^ ± 29.06	1050.00^**b**^ ± 0.00	1159.0 °C ± 27.63	1120.00^**bc**^ ± 31.62	1160.0 °C ± 18.44

Results are as Mean ± SE.

(Means with different superscripts within a row differ significantly, P < 0.001).

cTn-I is significantly higher in FMD-positive < 2 months calves showing clinical signs suggestive of myocarditis (A_1_:11.65 ± 0.34) as compared to FMD-positive < 2 months and > 2 months < 4 months calves showing no clinical signs suggestive of myocarditis (A_2_: 0.06 ± 0.00, B_1_:0.052 ± 0.002). The mean serum cTn-I of FMD negative < 2 months old (group C) and > 2 months and < 4 months old (group D) control group calves were 0.047 ± 0.01 and 0.047 + 0.00 respectively.

Likewise, mean serum AST is significantly higher in group A_1_ (264.83 ± 4.16) as compared to A_2_ (110.00 ± 0.00), B_1_ (83.00 ± 3.00), and control groups C (67.5 ± 3.10), and D (72.833 ± 2.09). In contrast to FMD-negative control groups of calves, FMD-positive calves across all age groups had significantly higher mean serum AST, regardless of clinical signs—positive or negative—suggestive of myocarditis.

Also, mean serum LDH is significantly higher in A_1_ (1213.33 ± 29.06) as compared to A_2_ (1050.00 ± 0.00), B_1_ (1159.00 ± 27.63), and FMD negative control group calves C (1120.00 ± 31.62) D (1160.00 ± 18.44).

However, the mean serum CK-MB level does not differ significantly between any of the groups.

### Necropsy and histopathological findings

**Figure 2:**
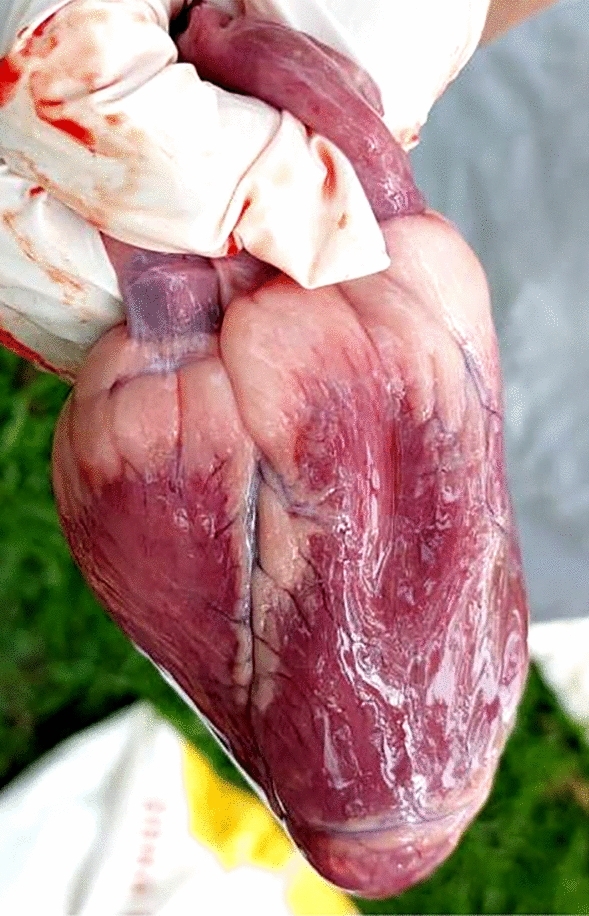
Gross image of < 2 months old calf heart showing pathognomic “tigroid heart” lesion.

**Figure 3:**
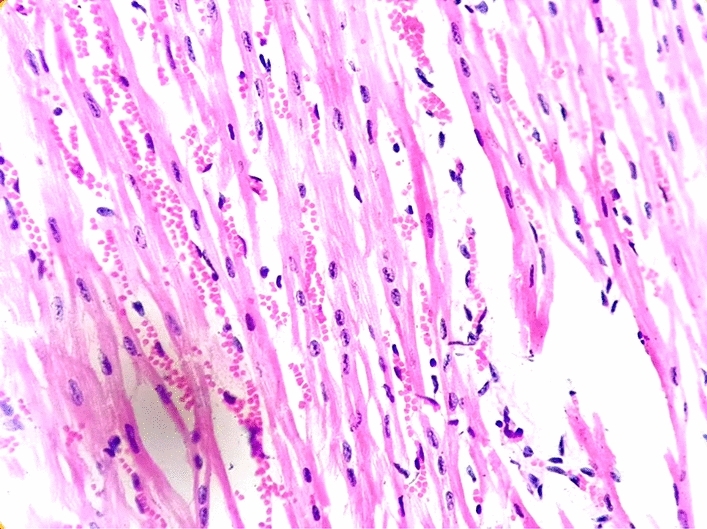
Microscopic image of calf heart showing coagulative necrosis of muscle fibers with interstitial infiltration of mononuclear cells. H&E.

At necropsy, the presence of a pathognomic gross lesion of an acute or myocardial form of FMD known as “tigroid heart” was observed as white streaks on the epicardium of the heart (Fig. 2). Histopathological examination revealed hyaline degeneration and necrosis of the myocardiocytes. The muscle fibers got separated from each other and there is a high degree of intramuscular hemorrhage. Striations of the muscle fibers are also lost. The muscle fiber of the heart tissue sections showed infiltration with lymphocytes and a few histiocytes (Fig. 3).

## Discussion

In the present study, the samples from FMD suspected calves were found to be positive for FMDV serotype “O” by Sandwich ELISA and RT-mPCR. This revealed the circulation of FMDV serotype "O" in Assam. Similar findings reported the dominance of serotype "O" as the cause of recent outbreaks in Assam were reported by^19^ and^20^.

FMD-associated myocarditis is fatal in young calves^21^. Cardiopulmonary pathological changes are the vital signs in FMD-infected calves. These vital signs can be assessed by physical examination of the heart and lungs. Clinical findings of fever, tachypnoea, tachycardia, and gallop rhythm have also been reported in FMD-infected young calves with myocarditis, as seen in this study^8,22^. One probable reason for these clinical findings may be attributed to the circulatory insufficiency caused due to myocarditis. Our study’s results agree with the previous investigations that recorded myocarditis in FMD-infected young calves under 2 months of age^8,9,23,24^. The affinity of FMDV to actively growing myocardial cells may be the cause of myocarditis due to FMD infection in young calves^9^. Studies report the entry of FMDV into heart cells is suspected to involve the attachment of the RGD loop of VP_1_ protein on viral capsid to the host integrins on the surface of the target cell^25,26^. Reference^27^ reported that age-associated changes occur in the cardiac matrix and integrins of the heart. Vesicular/ blistering lesions in the mouth and/ or feet are characteristic of FMD in adult cattle and acute severe myocardial injury in calves^7^. In the present study, 57.14% of FMD-infected calves under 2 months old (group A_0_) and all the calves aged > 2– < 4 months (group B_0_) showed the presence of vesicular lesions on the mouth and/or feet. Further, vesicular lesions were observed in 50% of the calves diagnosed as FMD positive with clinical signs suggestive of myocarditis (group A_1_) and in all calves diagnosed as FMD positive with clinical signs negative for myocarditis (group A_2_ and B_1_). Previous studies reported that FMDV myotropic may occur with or without vesicular lesions. Literature defines FMD-infected calves with the presence of both myocarditis and vesicles as expanded multi-tropism whereas the absence of vesicles as the substitution of tropism^8,28,29^. In the present study, 4 calves under 2 months old suspected clinically of myocarditis died. Previous studies also reported the sudden death of FMD-infected young calves due to damage to the myocardium^9^.

Serum AST, CK-MB, LDH, and cTn I were measured. Our study revealed a significant increase in mean serum cTn-I in FMD-infected calves (< 2 months) with clinical signs suggestive of myocarditis (A_1_: 11.65 ± 0.34) than those (< 2 months and > 2 months < 4 months) without clinically suggestive of myocarditis (A_2_: 0.06 ± 0.00 and B_1:_ 0.052 ± 0.002) and healthy control groups (C: 0.0466 ± 0.005 and D: 0.0466 ± 0.004). Our study’s results agree with^9,14^ and^22^. Mean serum AST is significantly higher in FMD-infected calves across all age groups, regardless of clinical signs suggestive of myocarditis or without myocarditis. Reference^9^ reported higher AST concentration in animals with myocarditis suggesting that increased AST level is an indication of heart abnormalities. Several studies report that the cTn-I marker is superior to other serum markers for the detection of myocyte injury in FMD-infected calves due to its higher cardiac specificity^9,14,29^. Therefore, from this study, it can be recognized that increased AST level concerning increased cTn I level is related to myocarditis in FMD-infected calves.

In agreement with^23^ necropsy of the dead calves revealed the presence of white streaks on the epicardium of the heart which is a pathognomic lesion known as “tigroid heart” in FMD-infected calves. Similar to^8^ and^23^ histopathological examination revealed myocardial degeneration and necrosis and infiltration of muscle fiber of the heart tissue sections with mononuclear cells predominated by lymphocytes with a few plasma cells and histiocytes.

During FMD outbreak, it is very common to find sudden death of young calves without premonitory signs in field conditions. In this scenario, biomarker assay in young calves may aid in depicting the actual status of cardiac function in FMDV-infected calves. The biomarkers cTn I, AST, CK-MB, and LDH are released into circulation as a response to myocardial injury or damage. Among these cTn I can be considered to be a sensitive marker of myocardial damage as compared to the enzymes AST, CK-MB and LDH because cTn I is the only troponin uniquely expressed in the myocardium. Consequently, this will assist in initiating appropriate therapeutic/ supportive measures and avert the related death.

## Conclusion

The primary investigation of the clinically FMD-suspected calves revealed the circulation of FMDV serotype “O” in Assam. All calves with myocarditis were under 2 months old, suggesting FMD-associated myocarditis is age dependent in suckling calves. Overall, this study reports the relevance of clinical and histopathological findings and biochemical markers in diagnosing FMD-related myocarditis in suckling calves.

### Supplementary Information

Supplementary Information.

## Data Availability

All data generated or analysed during this study are included in this published article [and its supplementary information files].

## References

[1] Grubman MJ, Baxt B (2004). Foot and mouth disease. Clin. Microbiol. Rev..

[2] Alexandersen S, Mowat N (2005). Foot-and-mouth disease: Host range and pathogenesis. Curr. Top. Microbiol. Immunol..

[3] Belsham GJ, Jamal SM (2018). Molecular epidemiology, evolution and phylogeny of foot-and-mouth disease virus. Infect. Genet. Evol..

[4] Sutmoller P, Casas Olascoaga R (2002). Unapparent foot and mouth disease infection (sub-clinical infections and carriers): Implications for control. Sci. Tech. Rev. World Organ. Anim. Health/Office Int. des Epizootie (OIE)..

[5] OIE. Old classification of diseases notifiable to the OIE (2019) http://www.oie.int/en/animal-health-in-the-world/the-world-animal-health-informationsystem/old-classification-of-diseases-notifiable-to-the-oie-list-a/. Accessed 22 July 2022

[6] Donaldson AI, Sellers RF, Martin WN, Aitken ID (2000). Foot-and-mouth disease. Diseases of sheep.

[7] Alexanderson S, Zhang Z, Donaldson AI (2003). The pathogenesis and diagnosis of foot-and-mouth disease. J. Comp. Pathol..

[8] Karapinar T, Dabak D, Kuloglu T (2010). High cardiac troponin I plasma concentration in a calf with myocarditis. Can. Vet. J..

[9] Aktas MS, Ozkanlar Y, Oruc E (2015). Myocarditis associated with foot and mouth disease in suckling calves. Veterinarski arhiv..

[10] Jaffe AS, Landt Y, Parvin CA (1996). Comparative sensitivity of cardiac troponin I and lactate dehydrogenase isoenzymes for diagnosing acute myocardial infarction. Clin. Chem..

[11] Weber M, Rau M, Madlener K (2005). Diagnostic utility of new immunoassays for the cardiac markers cTnI, myoglobin and CK-MB mass. Clin Biochem..

[12] O'Brien PJ (2008). Cardiac troponin is the most effective translational safety biomarker for myocardial injury in cardiotoxicity. Toxicology.

[13] Hastings KE (1997). Molecular evolution of the vertebrate troponin I gene family. Cell Struct. Funct..

[14] Dawood AA, Alsaad KM (2018). Clinical and diagnostic studies of myocarditis result from FMD in lambs. J. Agric. Vet. Sci..

[15] Zinnah MA, Islam MT, Rahman MM (2010). Standardization of multiplex reverse transcriptase polymerase chain reaction and typing of foot and mouth disease virus prevalent in Bangladesh. Bangl. J. Vet. Med..

[16] Borah B, deka P, Sharma K,  (2018). Isolation, identification and retrospective study of foot and mouth disease virus from affected Mithun (Bos frontalis) in north-eastern India. Transboun Emerg. Dis..

[17] Giridharan P, Hemadri D, Tosh C (2005). Development and evaluation of a multiplex PCR for differentiation of foot-and-mouth disease virus strains native to India. J. Virol. Meth..

[18] Slaoui M, Fiette L (2011). Histopathology procedures: from tissue sampling to histopathological evaluation. Methods Mol. Biol..

[19] Baro S, Sharma K, Borah B (2019). Molecular epidemiology and phylogenetic analysis of foot and mouth disease virus type ‘O’ and evaluation of its carrier state in cattle of Assam by Real time PCR. Indian J. Anim. Res..

[20] Das L, Sharma K, Borah P (2022). Detection of foot-and-mouth disease virus type O in recovered as well as healthy cattle to study carrier status in Assam. Indian J. J. Exp. Biol..

[21] Barker IK, Van Dreumel AA, Palmer N, Jubb KVF, Kennedy PC, Palmer N (1993). The alimentary system. Pathology of Domestic Animals.

[22] Tunca RM, Sozmen H, Erdogan M (2008). Determination of cardiac troponin I in the blood and heart of calves with foot-and-mouth disease. J. Vet. Diagn. Invest..

[23] Gunes V, Erdogan HM, Citil M (2005). Assay of cardiac troponins in the diagnosis of myocardial degeneration due to foot and mouth disease in a calf. Vet. Rec..

[24] Gulbahar MY, Dawis WC, Guvenc T (2007). Myocarditis associated with foot and mouth disease virus type O in lambs. Vet. Pathol..

[25] Jackson T, Sheprard D, Denyer M, Blakemore W, King AM (2000). The epithelial integrin avb6 is a receptor for foot and mouth disease virus. J. Virol..

[26] Jackson T, Mould AP, Sheppard D, King AM (2002). Integrin avb1 is a receptor for foot and mouth disease virus. J. Virol..

[27] Burgess ML, Mccrea JC, Hedrick HL (2001). Age–associated changes in cardiac matrix and integrins. Mech. Ageing. Dev..

[28] Arzt J, Baxt B, Grubman MJ (2011). The pathogenesis of foot-and mouth disease II: Viral pathways in swine, small ruminants, and wildlife; myotropism, chronic syndromes, and molecular virus host interactions. Transbound. Emerg. Dis..

[29] Sobhy NM, Yasmin HB, Sunil KM (2018). Outbreaks of foot and mouth disease in Egypt: Molecular epidemiology, evolution and cardiac biomarkers prognostic significance. Int. J. Vet. Sci. Med..

