# Supplementary material for: Foot-and-mouth disease-associated myocarditis is age dependent in suckling calves
Source: Sci Rep. 2024 May 4;14:10289. doi: 10.1038/s41598-024-59324-9 (PMC11069542; doi:10.1038/s41598-024-59324-9)
Supplement: Supplementary file 1 — Supplementary Information. [file 41598_2024_59324_MOESM1_ESM.pdf]

Supplementary Table 1: Serum biochemical findings of calves in different groups

Parameters	Age of the animals	FMD positive		Control (FMD Negative)
		Clinical sign positive	No clinical sign	
cTn-I [µg/L]	< 2months	11, 11, 12, 12, 10.9, 13 (11.65±0.34)	0.06	0.05, 0.03, 0.03, 0.06, 0.06, 0.05 (0.0466 + 0.005578)
	>2< 4 months	0	0.05, 0.05, 0.05, 0.06, 0.06, 0.05, 0.05, 0.04, 0.06, 0.05 (0.052±0.002)	0.03, 0.06, 0.04, 0.05, 0.05, 0.05 (0.0466+0.004216)
AST [U/L]	< 2months	260, 265, 275, 255, 280, 255 (264.833±4.16)	110	80, 60, 70, 70, 60, 65 (67.5±3.095695937)
	>2< 4 months	0	90, 70, 100, 80, 80, 90, 70, 80, 90, 80 (83±3)	70, 75, 80, 65, 75, 72 (72.833±2.088327348)
CK-MB [U/L]	< 2months	190, 200, 240, 260, 250, 260 (233.3333±12.56)	210	320, 280, 260, 190, 250, 240 (256.67±17.63834207)
	>2< 4 months	0	220, 300, 320, 280, 260, 190, 310, 240, 200, 240 (256±14.46835628)	220, 300, 220, 200, 240, 260 (240±14.60593487)
LDH [U/L]	< 2months	1280, 1250, 1250, 1100, 1250, 1150 (1213.33±29.05932629)	1050	1020, 1050, 1140, 1230, 1170, 1110 (1120±31.6227766)
	>2< 4 months	0	1080, 1100, 1250, 1340, 1180,	1150, 1240, 1160, 1100, 1160, 1150

			1200, 1100, 1050, 1140, 1150<br><br>(1159±27.62647683)	(1160±18.43908891)
